# Bilateral sleeve fracture of the inferior pole of the patella in children: A case report

**DOI:** 10.3389/fsurg.2022.970802

**Published:** 2022-09-23

**Authors:** Tangjiang Li, Min Xiang, Xin Lv, Yongqiao Gan, Song Yu

**Affiliations:** ^1^ Department of Pediatric Surgery, Affiliated Hospital of Zun Yi Medical University, Zunyi City, China; ^2^ Department of Pediatric Surgery, Guizhou Children's Hospital, Zunyi City, China; ^3^ Department of Electrocardiogram Room, Affliated Hospital of Zun Yi Medical University, Zunyi city, China; ^4^ Guizhou University of Traditional Chinese Medicine, Guiyang city, China

**Keywords:** child, bilateral patellar fracture, sleeve fracture, tension band wiring, pediatric orthopaedics

## Abstract

**Background:**

A patellar avulsion fracture is a unique fracture type in children with an extremely low incidence rate in knee joint injuries, most of which are unilateral. Cases of simultaneous bilateral fracture are rare. Due to the physiological and anatomical characteristics of patellar sleeve fractures in children, obvious avulsed bony fragments are seldom seen with plain radiography after trauma; thus, this fracture type is often misdiagnosed or missed on first evaluation. Early diagnosis of patellar sleeve fracture is the key to successful treatment.

**Case report:**

This paper presents a case of bilateral patellar sleeve fracture in a 10-year-old girl that was missed in another hospital and treated successfully by open reduction and fixation of the osteochondral fragments after the patient was transferred to our hospital, yielding good clinical outcomes at the one-year follow-up.

**Conclusion:**

Overall, this case report described the clinical and imaging characteristics of inferior pole sleeve fractures in children and highlight clinicians’ awareness of this injury to assist in early, accurate diagnosis and reduce the incidence of misdiagnosis and missed diagnosis due to unfamiliarity.

## Introduction

Patellar fractures in children account for 1% of all fractures in the pediatric population. Patellar sleeve fractures represent 57% of these injuries (1), and the majority are unilateral. Bilateral fractures are even rarer in a healthy child, with only a few cases reported in the literature. Patellar avulsion fractures in children often involve the lower pole of the patella in the form of an osteochondral avulsion that may be subtle on a plain radiograph, leading to missed diagnosis or underestimation of the injury’s severity. This is the case with the injury discussed in this report, which was originally misdiagnosed as a knee contusion at another hospital. Therefore, it is challenging to diagnose avulsion fractures in children, especially for junior doctors and doctors unfamiliar with knee fractures in this population. This paper has been written primarily as an interesting case report to draw attention to the difficulties of diagnosing and treating patellar sleeve fractures in children.

This study has been approved by the Medical Ethics Association of the Affiliated Hospital of Zunyi Medical University (approval number: KLL-2020-271). Informed consent was obtained from the patient before she was included in the study.

## Case report

**Figure 1:**
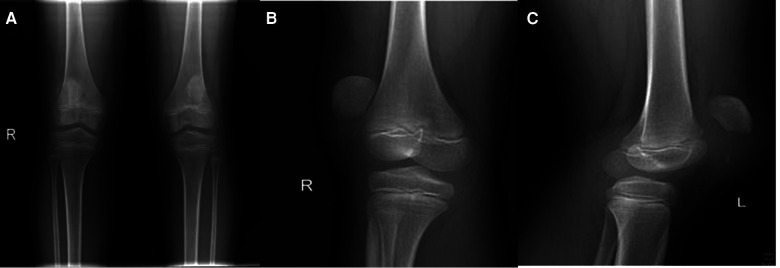
(**A**) AP radiograph of the both knees. (**B**) Lateral radiograph of the right knee. (**C**) Lateral radiograph of the left knee.

**Figure 2:**
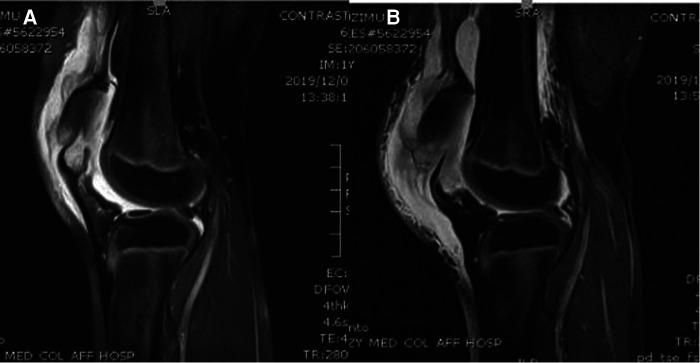
(**A**) Sagittal T2 MRI of the right knee. (**B**) Sagittal T2 MRI of the left knee.

A healthy 10-year-old female patient was transferred from another hospital to our hospital because of the knee pain she experienced after she fell on her knees while running. The initial clinical complaint was sudden and severe knee pain, with a subsequent inability to walk. In the teaching hospital she initially visited, she was diagnosed with contusion of both knee joints; later, she was referred to our hospital for treatment because of obvious pain and swelling and an inability to walk. She had no significant past medical, drug, or family histories. On physical examination, in addition to pain, swelling, and joint effusion of the bilateral knee joints, a palpable gap at the inferior border of the patellae was found on both sides, and she was unable to raise both lower limbs. The clinical examination of the head and neck, chest and abdomen, spine, and pelvis showed normal results. The radiographs of both knees showed soft tissue swelling and high-riding patellae, indicating sleeve fractures (Figures 1A–C). Magnetic resonance imaging (MRI) was then performed to confirm the diagnoses of quadriceps tendon tear and patellar sleeve fracture and better reveal the extent of the displaced bony fragments (Figures 2A,B). Therefore, isolated and simultaneous bilateral sleeve fractures of the patellae were diagnosed based on clinical signs of extensor mechanism disruption (high-riding patellae and a gap at the lower pole of each patella) and imaging studies. Surgical intervention was performed the day after the diagnosis, with open reduction and internal fixation technique management.

**Figure 3:**
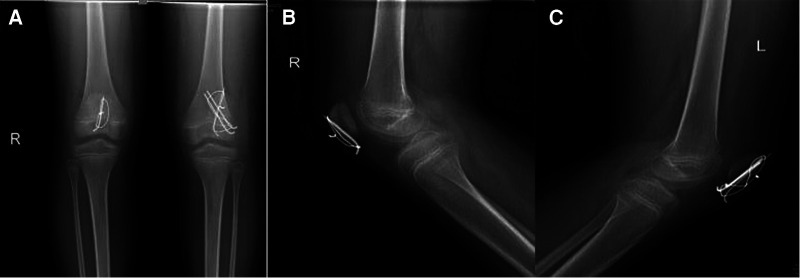
(**A**) Post-operative AP radiograph of the both knees. (**B**) Post-operative lateral radiograph of the right knee. (**C**) Post-operative lateral radiograph of the right knee.

During the surgery, the patient lay in the supine position under general anesthesia, and a tourniquet was applied on the proximal thigh. A midline incision was performed. The paratenon was incised at the midline and dissected off the proximal few centimeters of the proximal patellar tendon, and the fracture site was defined. To clear the debris and hematoma, both knees underwent a copious lavage. Tearing of the medial and lateral patellar retinacula was noted. There were no loose fragments in either knee joint. One-third of the articular cartilage of the patella and a corresponding portion of the patellar retinaculum were avulsed from the distal patella on the left side, and a small bony fragment was avulsed from the distal patella on the right side. The fracture on the left side was reduced and held with a patellar clamp, and the articular surface was evaluated for any malreduction. Three 2.0-mm Kirschner wires were used to maintain the reduction. An 18-gauge stainless-steel wire was placed across over the anterior surface of the patella with a figure-of-eight suture. The wires were sequentially tightened to apply equal tension across the fracture site. On the right side, because the bony fragment was too small to be fixed by two Kirschner wires, only one 2.0-mm Kirschner wire was used to fix the distal fracture of the patella, and an 18-gauge stainless-steel wire was used to encircle the patella. Then, tension banding was performed, and compression was achieved at the fracture site. The full range of patellar movement was assessed, and normal patellar tracking was noted. The wound was washed thoroughly and closed after the retinaculum was repaired. The postoperative radiographs are shown in Figures 3A–C.

**Figure 4:**
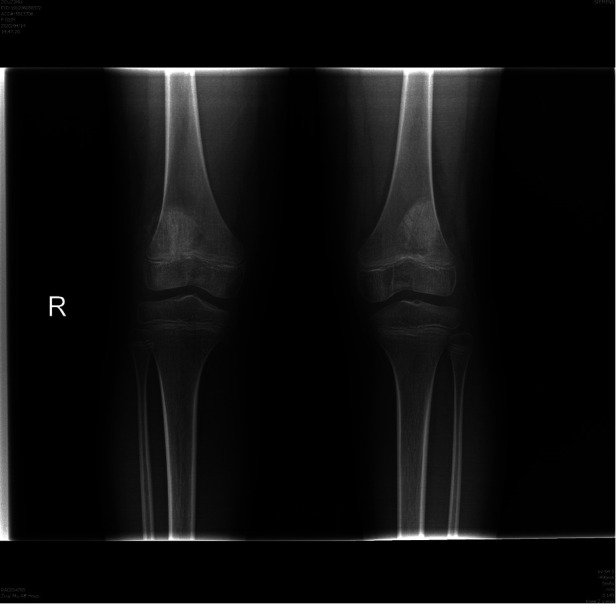
Post-operative AP radiograph of the both knees after removal of Kirschner and cerclage wires.

After the operation, both knees were immobilized with a fully extended cylinder cast for four weeks with non-weight-bearing, followed by a hinged knee brace for four weeks, during which the knees’ range of motion and weight-bearing status were gradually increased. Isometric quadriceps strengthening exercises were started after the surgery as well. The patient underwent a continuous rehabilitation program and progressively regained her knee movement. Gradual weight-bearing exercises with crutches were started at four weeks, and the patient regained full weight-bearing status without crutches at eight weeks. At the six-month follow-up, both knees were free of swelling, had a normal appearance, and could move within the full range of motion during free walking. The Kirschner and cerclage wires were removed 10 months after the surgery. The x-rays images after removal of Kirschner and cerclage wires are shown in Figure 4. The patient’s outcome was excellent, without complaints or symptoms of patellar instability and with normal muscle strength and a full range of motion in daily.

## Discussion

Patellar fractures in skeletally immature patients are rare, and patella sleeve fractures were first described by Houghton and Ackroyd in 1979 (2). A sleeve fracture is defined as an avulsion of a small bony fragment from the distal pole of the patella, along with its articular cartilage, periosteum, and retinacula, which are pulled away from the main body of the structure (3). The incidence of patellar sleeve fracture in children is much lower than that in adults, possibly due to the unique anatomical characteristics of the knee joint. Children have more flexible ligaments, and their joint capsules are relatively laxer than those of adults, which may protect the immature patella from injury. Furthermore, the patella is mostly composed of protective cartilage, making it more resistant (or resilient) to fractures (4, 5). Based on their location, avulsion fractures can be classified into four types, as described by Grogan et al.: (1) superior, which are the least common; (2) inferior, which are the most common and usually associated with an acute injury; (3) medial, which can lead to lateral dislocation of the patella; and (4) lateral, which are chronic stress lesions resulting from repetitive tensile pull from the vastus lateralis muscle (6).

Early and accurate diagnosis of inferior pole sleeve fractures is a challenge because the cartilage and periosteum may not be visible on plain radiographs. Thus, diagnosis of this injury at the initial presentation may be difficult or delayed, just as in the case reported here. Delayed diagnosis can lead to elongation of the patella, quadriceps muscle weakness, and permanent disability (2). Eliciting a detailed mechanism of injury and performing a thorough clinical and radiological examination are important diagnostic steps. Physical examination may be difficult when the knees are very tender and large effusion is present; thus, palpation of a high-riding patella may be all that is ascertained. Along with point tenderness, a palpable gap may be felt at the inferior pole of the patella if the fracture is obviously displaced (1, 3). If a sleeve fracture is suspected, imaging modalities might be helpful. Radiographic features of a high-riding patella and visible bony fragments may be present, and a diagnosis can be made; however, if the avulsed fragment has minimal bone within it, it can be easily missed on a radiograph (7, 8). In particular, junior physicians unfamiliar with this injury may miss or delay the diagnosis when a child presents with hemarthrosis without a bony fragment on x-ray. Bates (9, 10) recommended using MRI to assist in the diagnosis and management of sleeve fractures when the plain radiograph looks normal. Magnetic resonance imaging is helpful in assessing the need for surgery because it comprehensively demonstrates the extent of cartilaginous injury, joint involvement, and quadriceps tendon rupture. Although diagnosis may be challenging in the presence of pain and hemarthrosis, an awareness of the injury together with characteristic radiological features should raise suspicions of patellar fracture.

Patellar sleeve fracture treatment depends on the type of fracture, integrity of the extensor mechanism, and congruity of the articular surface. The indications for nonoperative treatment for certain types of patellar fractures include minimal (<2 mm) displacement, an intact articular surface, and a functionally intact extensor mechanism (11, 12). However, there is an argument for conservative management if there is a visible bony fragment on x-ray and <2 mm of displacement. If a bony fragment is visible on the radiograph and the displacement is minimal, conservative treatment with cylinder cast immobilization with a fully extended knee joint position is indicated. A nondisplaced or minimally displaced fracture may be displaced secondarily to the pull of the quadriceps muscle and might require surgery (13), so timely follow-up should be performed in conservative patients to determine whether the fracture is displaced. Surgical intervention is recommended for patellar fractures with >2 mm of displacement, comminuted fractures with articular surface disruption, and osteochondral fractures with comminution or displacement. Surgery should aim for anatomical reduction and reconstruction of the extensor mechanism, with stable fixation. Various open reduction and internal fixation techniques have been described in the literature, including tension banding with wires or cannulated screws, the Mason–Allen technique, Krackow whipstitches, bone anchors, and Bunnell whipstitches with screw fixation (6, 14, 15). The method selected is generally dependent on the surgeon’s personal preference and the bony fragments present. If surgery is performed properly and without delay, all methods can achieve satisfactory outcomes, just as in the case reported here. For this patient, on the left side, open reduction and internal fixation with tension band wiring were performed; on the right side, because the bony fragment was too small to be fixed by two Kirschner wires, only one 2.0 mm Kirschner wire was used to fix the distal fracture of the patella, and an 18-gauge stainless-steel wire was used to encircle the patella. Then, tension banding was performed, and compression was achieved at the fracture site. The outcome was found to be excellent for both knees at the one-year follow-up. However, in cases where the diagnosis and treatment are delayed or a displaced sleeve fracture is misdiagnosed and managed nonoperatively, the outcome is unsatisfactory. An untreated case may lead to complications such as patella magna, extensor lag, quadriceps muscle atrophy, or even permanent disability (16, 17).

## Conclusion

Patellar sleeve fractures are relatively rare in children, and bilateral cases are even rarer. Identification of this condition is essential; the diagnosis is often missed both clinically and radiologically since the distal bony fragment may be too small to detect by radiography. To avoid this issue, it is important to recognize this type of injury and its characteristics. Thus, this case report highlighted the characteristics of patellar sleeve fracture in a child to improve the understanding of this injury in children and reduce the rate of missed diagnosis and misdiagnosis.

## Data Availability

The original contributions presented in the study are included in the article/Supplementary Material, further inquiries can be directed to the corresponding author/s.
